# The Association of Apparent Myelin Water Fraction With Diffusional Kurtosis and Biophysical Modeling Parameters

**DOI:** 10.1002/nbm.70377

**Published:** 2026-08-27

**Authors:** Hunter G. Moss, Michael A. Sugarman, Jongho Lee, Andrew A. Chen, Jens H. Jensen, Andreana Benitez

**Affiliations:** ^1^ Center for Biomedical Imaging Medical University of South Carolina Charleston South Carolina USA; ^2^ Department of Neuroscience Medical University of South Carolina Charleston South Carolina USA; ^3^ Department of Pediatrics Medical University of South Carolina Charleston South Carolina USA; ^4^ Department of Neurology Medical University of South Carolina Charleston South Carolina USA; ^5^ Department of Electrical and Computer Engineering Seoul National University Seoul South Korea; ^6^ Department of Public Health Sciences Medical University of South Carolina Charleston South Carolina USA; ^7^ Department of Radiology Medical University of South Carolina Charleston South Carolina USA

**Keywords:** diffusional kurtosis imaging, fiber ball imaging, myelin water fraction, myelin water imaging, ViSTa‐MWI, white matter

## Abstract

Diffusion MRI (dMRI) methods including diffusion tensor imaging (DTI) and more advanced models including diffusional kurtosis imaging (DKI) and fiber ball white matter modeling (FBWM) probe various aspects of white matter (WM) microstructure. However, the associations between these variables and myelin content remain largely unknown. We examined this issue using ViSTa myelin water imaging (MWI), which estimates the proportion of tissue water associated with myelin using the apparent myelin water fraction (aMWF) and examined correlations with dMRI variables across heterogeneous deep WM regions. Four healthy adults underwent anatomical MRI, multishell dMRI, and ViSTa‐MWI. We computed DTI, DKI, FBWM, and aMWF metrics within a binarized deep WM atlas and callosal subregions (the genu and splenium) and evaluated associations between dMRI and aMWF using voxel‐wise block‐permutation Spearman correlations. Across WM regions, axonal water fraction (*ρ* = 0.68), mean kurtosis (*ρ* = 0.63), and radial kurtosis (*ρ* = 0.62) had stronger correlations with aMWF than conventional DTI metrics. Correlations for dMRI variables were consistently higher within the splenium (mean |*ρ*| = 0.68) compared to the genu (mean |*ρ*| = 0.56), reflecting stronger associations with aMWF in an earlier‐myelinating region with denser axonal packing and reduced fiber dispersion. DTI, DKI, and FBWM variables all correspond with aMWF. Select DKI and FBWM variables demonstrated the strongest associations with aMWF and were more sensitive than conventional DTI metrics. Correlations for all metric families were stronger within the splenium compared to the genu, presumably reflecting the high density of myelination in this region. These results help to clarify the biophysical interpretation of dMRI microstructural parameters by determining how strongly they are influenced by myelin content. This preliminary examination helps to clarify the roles of dMRI variables for future studies of WM integrity and reinforces the use of DKI and FBWM.

## Graphical abstract

We investigated associations between a panel of diffusion MRI metrics and myelin content, using ViSTa apparent myelin water fraction (aMWF) as a proxy for myelin. Fractional anisotropy (FA) showed a robust association with aMWF; however, more advanced metrics including radial kurtosis (*K*_⊥_) and axonal water fraction (*f*) had stronger correlations. Correlations were consistently stronger in the splenium than the genu, indicating that denser, earlier‐myelinating tracts better reveal myelin‐diffusion associations.

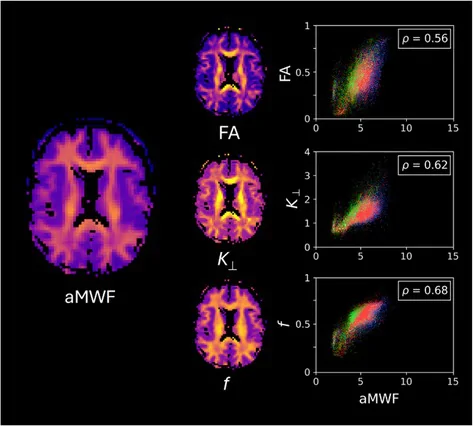

## 1 Introduction

Diffusion MRI (dMRI) provides information regarding microstructural characteristics of white matter (WM), including from conventional diffusion tensor imaging (DTI) [1] and more advanced metrics, including diffusional kurtosis imaging (DKI) [2] and biophysical modeling [3, 4, 5, 6, 7]. Yet, the biological correspondence of dMRI parameters remains uncertain [8, 9]. In particular, it is crucial to determine the associations between dMRI metrics and myelin content, given myelin's central role in WM microstructural organization [10, 11, 12]. Examining these associations in healthy adults can help to clarify which dMRI metrics are most influenced by myelin content and facilitate their interpretation as indices of microstructure in clinical populations.

Conventional DTI estimates the diffusion tensor to generate parameters such as fractional anisotropy (FA), mean diffusivity ($\bar{D}$), axial diffusivity (*D*_∥_), and radial diffusivity (*D*_⊥_) to summarize water directionality and mobility within cortical microstructure, including WM [1]. A limitation of DTI is its assumption of a Gaussian distribution of water displacement, which oversimplifies the intricate tissue microenvironment. DKI extends DTI by allowing for non‐Gaussian diffusion dynamics, providing kurtosis metrics that are sensitive to tissue complexity and barriers such as myelin [2]. However, both DTI and DKI interpretations are constrained by their lack of compartmental specificity, as they aggregate diffusion signals arising from intra‐axonal, extra‐axonal, and free‐water compartments.

To address this limitation, Fiber Ball Imaging (FBI) [3] uses large *b*‐values (≥ 4000 s/mm^2^) to isolate the intra‐axonal water signal, and assumes axons are thin, impermeable, straight cylinders [13, 14, 15]. The primary quantity derived with FBI is the fiber‐orientation density function (fODF) estimated from a straightforward linear transform of high *b*‐value dMRI data. The fODF allows for the calculation of parameters such as fractional anisotropy axonal (FAA) [14], the intra‐axonal analog of FA from DTI. The Fiber Ball White Matter model (FBWM) extends FBI by integrating lower *b*‐value dMRI (e.g., DTI) to incorporate extra‐axonal Gaussian diffusion [4]. As a biophysical model, FBWM estimates additional parameters specific to intra‐ and extra‐axonal WM compartments, including the axonal water fraction (f), intra‐axonal diffusivity (*D*_*a*_), and extra‐axonal mean, axial, and radial diffusivities (${\bar{D}}_{e}$, *D*_*e*,∥_, *D*_*e*,⊥_). These tissue‐specific biophysical metrics show regional sensitivity to age‐related microstructural alterations in the genu and splenium of the corpus callosum (gCC and sCC, respectively) [16]. However, the associations between these metrics and myelin content have not yet been evaluated.

There are multiple methods for estimating myelin content using MRI. Conventional myelin water imaging (MWI) is one method that estimates the myelin water fraction (MWF) by calculating the proportion of short‐T_2_ signal attributable to water trapped between myelin lipid bilayers [17]. The MWF is a suitable myelin content surrogate, as strong correlations have been observed with histological myelin staining [18, 19, 20, 21]. Multicomponent T2‐based MWF mapping has historically required relatively long acquisition times (12–15 min) [22]; however, modern implementations (e.g., accelerated 3D‐GRASE and compressed‐sensing multispin‐echo approaches) can achieve whole‐brain MWF in around 8 min [23, 24]. Visualization of short transverse relaxation time component (ViSTa) [25] is an alternative MWI technique based on the short‐T_1_ myelin water signal that generates an apparent MWF (aMWF) with a total scan time of 7–8 min. Although aMWF values are approximately 1/3 of conventional MWF, it nonetheless demonstrates high reproducibility and sensitivity to demyelination in clinical populations, including multiple sclerosis and neuromyelitis optica spectrum disorder [26, 27, 28, 29]. Thus, aMWF is a reasonable surrogate for myelin content in WM.

Prior studies have found significant associations between conventional MWF and DTI metrics [30, 31, 32, 33, 34], with FA and *D*_∥_ demonstrating the strongest correlations with MWF. However, these associations have not yet been examined using the aMWF from ViSTa. Additionally, no study to this point has evaluated how advanced metrics including DKI, FBI, and FBWM metrics correspond with myelin content. Thus, this is the first attempt to determine the associations between a panel of advanced dMRI methods and aMWF in WM. We approached this study using within‐person voxel‐wise correlations across dMRI contrasts in a sample of healthy young adults to first identify WM characteristics and dMRI metrics with the most consistent associations, so that future hypotheses and inferences can be refined when using these metrics to indicate changes in myelin content in aging or disease. We examined associations across a range of heterogeneous deep WM structures along with preselected WM ROIs with well‐established microstructural differences (i.e., the gCC and sCC) to determine how associations differ based on the degree of myelin content.

We hypothesized that: (1) aMWF would exhibit robust correlations with conventional DTI variables including FA and *D*_∥_, replicating prior findings with MWF [30, 31]; (2) DKI and FBWM variables suspected to be associated with myelin content such as radial kurtosis (*K*_⊥_), *f*, FAA, and extra‐axonal axial diffusivity (*D*_*e*,∥_) would display the strongest correlations with aMWF across regions; and (3) associations between dMRI metrics and aMWF would be more prominent in regions with higher myelin content such as the sCC compared to the gCC. These results will refine the interpretation of dMRI parameters as indicators of myelin content in healthy adults and inform future studies examining development, aging, and clinical populations.

## 2 Methods

### 2.1 Participants and MRI Acquisition

Four healthy adult volunteers (aged 33–34 years) provided written informed consent under an IRB‐approved protocol at the Medical University of South Carolina. We acquired imaging data using a 3 T Prisma^fit^ MRI scanner (Siemens Healthineers, Erlangen, Germany), including anatomical T1‐weighted images, ViSTa‐MWI, and multishell dMRI.

T1‐weighted imaging was conducted employing a 3D magnetization‐prepared rapid gradient echo (MP‐RAGE) sequence with the following parameters: echo time (TE) = 2.26 ms, repetition time (TR) = 2300 ms, inversion time (TI) = 900 ms, isotropic voxel size = 1 mm^3^, field of view (FOV) = 256 × 256 mm^2^, parallel imaging factor = 2, and total scan duration of 5 min and 21 s.

MWI data were obtained using a ViSTa‐based multiecho gradient echo (GRE) sequence with double inversion preparation [29]. The scan parameters included 56 slices, FOV = 192 × 192 mm^2^, isotropic voxel size = (2 mm)^3^, TR = 1160 ms, TI1 = 560 ms, TI2 = 220 ms, delay time (TD) = 380 ms, TE = 5.5 ms (echo spacing = 0.93 ms), 15 echoes, flip angle = 90°, matrix = 96 × 96, bandwidth = 1336 Hz/Px, partial Fourier factor = 6/8, and scan duration of 7 min and 40 s. To compute the apparent myelin water fraction (aMWF), a second multiecho GRE sequence was acquired without inversion pulses, utilizing identical parameters except for TR = 100 ms and flip angle = 28°, taking only 43 s to acquire.

Diffusion‐weighted images were captured employing a bipolar diffusion‐weighted echo planar imaging (EPI) sequence. Two acquisition schemes were employed: (1) *b*‐values of 0, 1000, and 2000 s/mm^2^ with 30 diffusion directions each, and (2) *b*‐values of 0 and 6000 s/mm^2^ with 128 directions. Parameters common across *b*‐values were: TE = 95 ms, TR = 4800 ms, isotropic voxel size = (3 mm)^3^, 42 axial slices, FOV = 222 × 222 mm^2^, full‐Fourier encoding, bandwidth = 1648 Hz/Px, and echo spacing = 0.69 ms. An additional 10 *b* = 0 s/mm^2^ volumes and a reverse‐phase encoding image were obtained. Total scan durations: (1) 6 min and (2) 11 min and 30 s.

### 2.2 Image Processing and Modeling

We processed ViSTa‐MWI data using an in‐house Python implementation to generate aMWF maps. By design, ViSTa aMWF is approximately 1/3 the magnitude of conventional MWF and not intended to determine absolute myelin quantification. However, aMWF still closely corresponds with myelin content and is suitable for examining the current hypotheses [25].

We used PyDesigner [35], a Python‐based version of the DESIGNER v1.0 pipeline [36], to complete dMRI data preprocessing, including MP‐PCA denoising [37], Gibbs ringing correction [38], motion and eddy current correction [39], and Rician bias correction [40]. Afterwards, it also produced the diffusion and kurtosis tensors computed from a DKI analysis using *b* = 0, 1000, and 2000 s/mm^2^ [2]. Conventional DTI metrics included mean, axial, and radial diffusivity ($\bar{D}$, *D*_∥_, *D*_⊥_) and fractional anisotropy (FA). DKI metrics included mean, axial, and radial kurtosis ($\bar{K}$, *K*_∥_, *K*_⊥_), and kurtosis FA (KFA).

Using the *b* = 0 and 6000 s/mm^2^ dMRI shells, we performed an FBI analysis to estimate the fODF in each WM voxel. From the fODF, a spherical harmonic expansion in even degrees was performed up to and including *l =* 8. We calculated FAA from the lowest two degrees (*l* = 0 and 2) [3, 14]. We also included the Matusita anisotropy axonal (MAA), a generalization of the FAA that utilizes all harmonic expansion degrees and better highlights the contributions of high angular frequency components [41]. We next constructed the FBWM model [4] by supplementing FBI data with the diffusion tensor estimated from DKI. As a biophysical model, FBWM estimates f, along with additional compartment‐specific parameters: intra‐axonal diffusivity (*D*_*a*_), extra‐axonal mean, axial, and radial diffusivities (${\bar{D}}_{e}$, *D*_*e*,∥_, *D*_*e*,⊥_), and extra‐axonal FA (FAE). Table 1 lists abbreviations and brief descriptions of all imaging metrics for reference. See Figures S1 and S2 for visualization of parameter maps.

**TABLE 1:** Reference for symbols and metrics in the analysis.

Symbol	Metric	Interpretation
aMWF	Apparent myelin water fraction	Fraction of water associated with myelin; higher ≈ more myelin
$\bar{D}$	Mean diffusivity	Average water mobility; higher often reflects less restriction/more free water.
*D*_∥_	Axial diffusivity	Diffusivity along primary fiber direction; sensitive to axonal integrity/packing
*D*_⊥_	Radial diffusivity	Diffusivity perpendicular to fibers; often increases with demyelination/less restriction
FA	Fractional anisotropy	Degree of directional diffusion; higher in coherent, aligned fiber bundles
$\bar{K}$	Mean kurtosis	Overall non‐Gaussian diffusion; higher implies greater tissue complexity
*K*_∥_	Axial kurtosis	Non‐Gaussianity along fibers; sensitive to longitudinal restrictions/heterogeneity
*K*_⊥_	Radial kurtosis	Non‐Gaussianity across fibers; reflects transverse barriers (e.g., myelin)
KFA	Kurtosis fractional anisotropy	Anisotropy of kurtosis; complements FA by emphasizing complexity anisotropy
*f*	Axonal water fraction	Relative intra‐axonal water contribution; higher ≈ denser axonal packing
*D*_a_	Intra‐axonal diffusivity	Intra‐axonal diffusivity; reflects diffusion within axons under model assumptions
FAA	Fractional anisotropy axonal	Intra‐axonal analog of FA; coherence of axonal orientations (high *b* signal)
MAA	Matusita anisotropy axonal	Like FAA but includes higher angular detail; sensitive to fine orientation structure
${\bar{D}}_{e}$	Extra‐axonal mean diffusivity	Mean extra‐axonal diffusivity; average mobility in extra‐axonal space
*D*_*e*,∥_	Extra‐axonal axial diffusivity	Extra‐axonal diffusivity along fibers; sensitive to extra‐axonal geometry/packing
*D*_*e*,⊥_	Extra‐axonal radial diffusivity	Extra‐axonal diffusivity across fibers; sensitive to transverse hindrance/barriers
FAE	Fractional anisotropy extra‐axonal	Extra‐axonal anisotropy; directional organization of diffusion in extra‐axonal space

*Note:* All diffusivities have units [μm^2^/ms].

### 2.3 Image Registration and ROI Definition

We skull‐stripped all MR images using FSL's Brain Extraction Tool [42]. We then registered all MR images to each subject's mean *b* = 0 s/mm^2^ image as the reference via FSL's Linear Image Registration Tool [43, 44] with “corratio” cost function and trilinear interpolation. Next, we registered the 1 mm MNI brain to each subject's T1 space using an affine transformation, which was then concatenated with the T1 to native diffusion space transform. We applied this combined transform to the binary MNI cerebellum mask and the Johns Hopkins University (JHU) ICBM‐DTI‐81 WM atlas [45] with nearest‐neighbor interpolation to bring them into native diffusion subject space.

The ROI for WM analysis was defined as the binarized version of the entire JHU WM atlas. This atlas prioritizes major deep white matter tracts that are reliably identifiable in diffusion space with coherent orientation [46]. In doing so, it omits superficial and short‐range peripheral tracts that have greater variability and are more challenging to replicate across participants due to issues including partial‐volume effects and complex fiber architecture [47]. Thus, although this ROI was not a comprehensive survey of all WM regions, it permitted an analysis of reliably defined regions with heterogeneous myelin content to determine the associations of interest. Any voxels overlapping the cerebellum were excluded to ensure WM homogeneity. To mitigate CSF partial‐volume effects, voxels with $\bar{D}$ > 1.5 μm^2^/ms were also excluded.

Within WM, we also wanted to investigate regional heterogeneity based on structures with established differences in microstructural characteristics (Hypothesis 3). Thus, we extracted the gCC and sCC from the JHU WM atlas for further region‐specific analyses. These ROIs were specifically selected to compare regions with variable myelination, axon thickness, and fiber‐orientation dispersion to determine potential discrepancies across these well‐defined intrahemispheric WM tracts. The three ROIs (i.e., WM, gCC, and sCC) thus enabled simultaneous investigation across diverse WM regions, with additional analyses in regions with predefined characteristics. On average, extracted ROIs contained 3958 ± 550 voxels for WM, 234 ± 43 voxels for the gCC, and 323 ± 82 voxels for the sCC (subject‐level values provided in Table S1).

Within each ROI, we calculated signal‐to‐noise ratio (SNR) as the ratio of the average dMRI signal in the *b*‐value shell at 6000 s/mm^2^ to the background noise estimated during the denoising preprocessing step, multiplied by (π/2)^1/2^; SNR values, on average, in the extracted ROIs were 8 ± 1 (WM), 6 ± 1 (gCC), and 8 ± 1 (sCC) with subject‐level values provided in Table S2. Because these data were reconstructed from multichannel coil images and repeated measurements were not acquired, these values should be interpreted as approximate rather than absolute SNR measurements.

### 2.4 Statistical Analysis and Hypothesis Testing

Significance for all hypotheses was assessed using block‐permutation, which preserves spatial dependence. Cubic blocks were sized to the maximum per‐axis half‐width‐half‐max (HWHM) (≈6 mm for WM and ≈9 mm for gCC and sCC, respectively). Two‐sided *p*‐values (significance set at *p* < 0.05) used an add‐one correction; multiplicity was controlled via max‐statistic family‐wise‐error rate (FWER). We then pooled the voxels across participants to calculate the average correlation between aMWF and each dMRI metric for all three ROIs. We characterized correlation strength as very weak (*ρ* < 0.2), weak (0.2 ≤ *ρ* < 0.4), moderate (0.4 ≤ *ρ* < 0.6), strong (0.6 ≤ *ρ* < 0.8), and very strong (*ρ* ≥ 0.8).

We then examined correlations between dMRI metrics and aMWF in a manner consistent with our three main hypotheses. (1) To determine associations between aMWF and conventional diffusion indices across WM tracts, we examined correlations between aMWF and DTI metrics (i.e., FA, $\bar{D}$, *D*_∥_, and *D*_⊥_) within the WM ROI. (2) To compare associations between advanced and conventional dMRI metrics, we examined analogous voxel‐wise correlations between aMWF and DKI/FBI/FBWM metrics in WM. (3) To determine how associations between dMRI and aMWF vary across WM regions with predefined characteristics, we examined voxel‐wise correlations between all dMRI metrics and the callosal ROIs in the sCC and gCC.

## 3 Results

**FIGURE 1:**
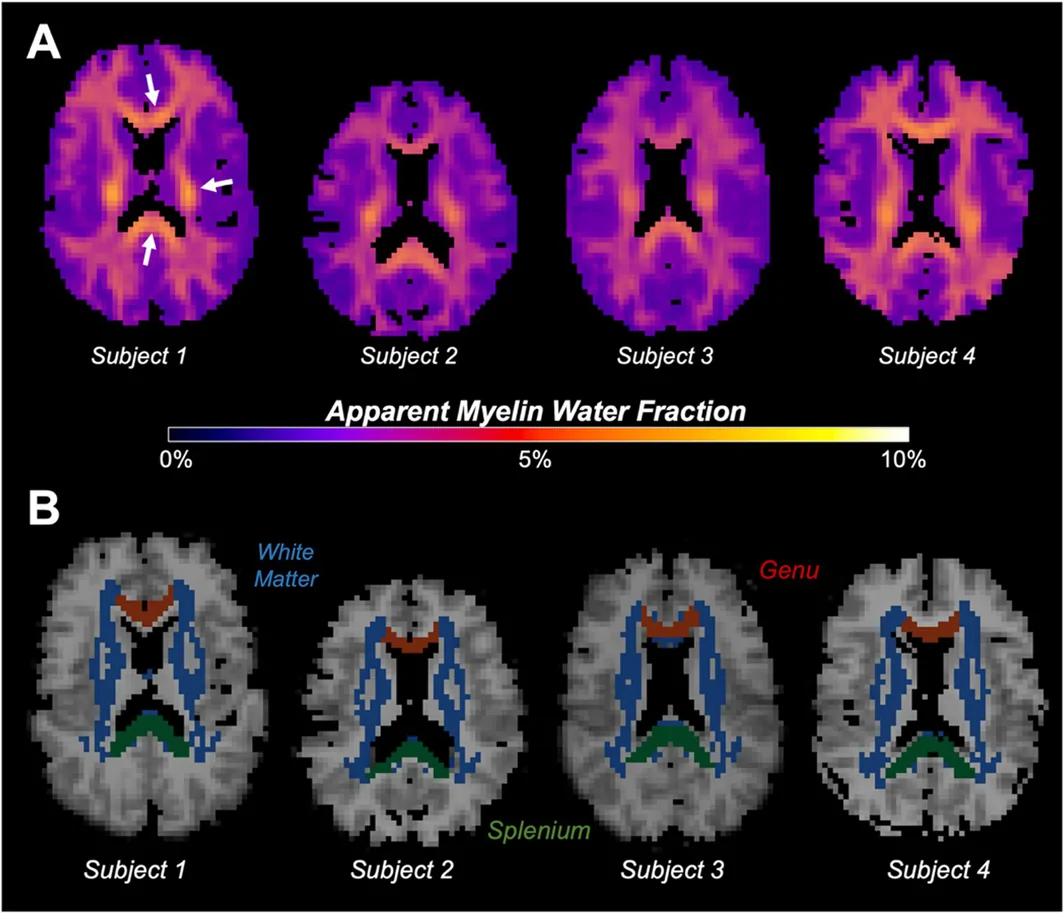
(A) Apparent myelin water fraction (aMWF) maps for all subjects. Arrows on Subject 1 indicate high aMWF areas in the genu, splenium, and posterior limb of the internal capsule. (B) White matter regions of interest (ROIs) for analyses. The primary axial image utilized is the *b* = 0 s/mm^2^ image for each subject, which was overlaid with the entire binarized Johns Hopkins white matter atlas for white matter tracts (blue), including the genu (orange) and splenium (green) regions of the corpus callosum.

**FIGURE 2:**
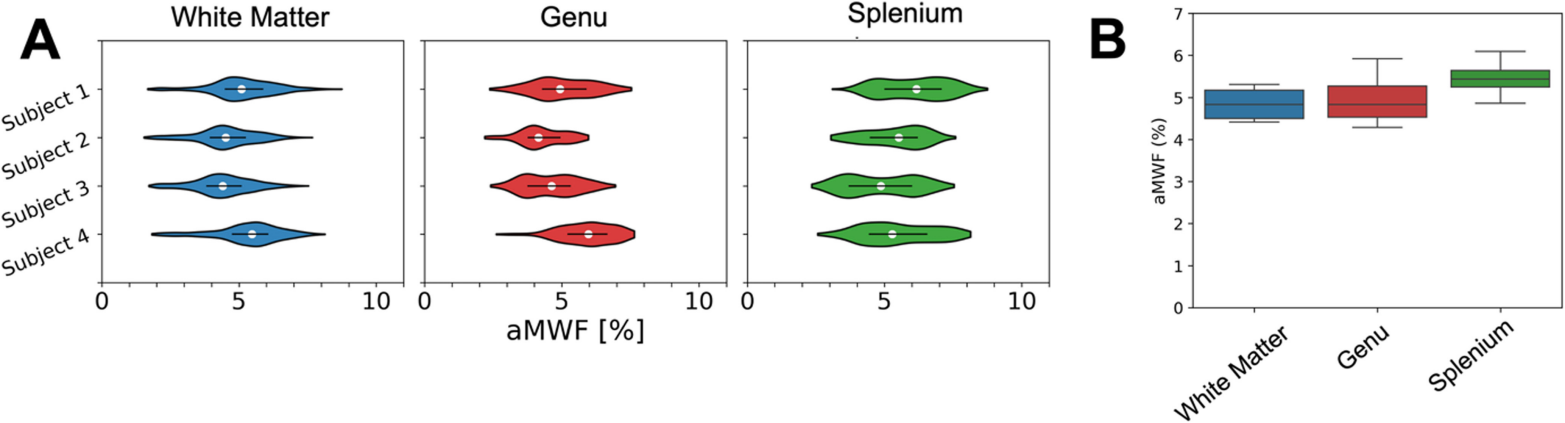
(A) Violin plots illustrating the apparent myelin water fraction (aMWF) for individual subjects within each region of interest. The white dot indicates the median aMWF value, whereas the black bar within the distribution depicts the interquartile range. (B) Group aMWF values.

Visual inspection of aMWF maps revealed consistent spatial patterns across subjects, with the highest values in WM regions with dense axonal packing and substantial myelin content including the sCC and posterior limb of the internal capsule (Figure 1A). Comparatively, regions with less coherent fiber bundles or heavy crossings showed lower aMWF. Quantitative analysis within selected ROIs (Figure 1B)—WM, the gCC, and the sCC illustrated variability both across subjects and regions (Figure 2A). Average aMWF was higher in the sCC (5.45%) compared to the gCC (4.97%) and WM (4.84%; Figure 2B and subject‐level provided in Table S3). dMRI metric values for each participant and across the group are provided in Table S4 and Figure S3.

**FIGURE 3:**
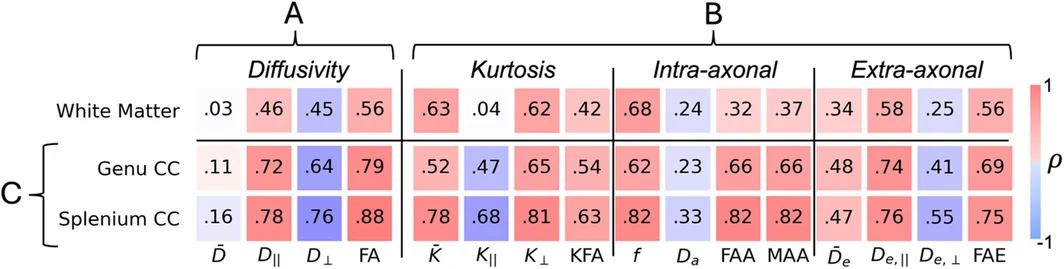
Voxel‐wise Spearman correlation coefficients (*ρ*) for the apparent myelin water fraction (aMWF) with dMRI metrics, including diffusivity, kurtosis, and biophysical parameters presented in intra‐ and extra‐axonal white matter. Correlations were calculated for each subject within WM from the binarized Johns Hopkins University WM atlas, along with specific ROIs in the splenium and genu of the corpus callosum. The mean correlation is shown, pooled from voxels across the four participants. Positive and inverse correlations are marked by red and blue squares, respectively, with shading indicating the strength of the correlation. (A) Analyses for Hypothesis 1, examining correlations between aMWF and conventional diffusion metrics in WM. (B) Analyses for Hypothesis 2, examining correlations between advanced kurtosis, intra‐axonal, and extra‐axonal diffusivity metrics in WM. (C) Analyses for Hypothesis 3, comparing correlations between dMRI metrics and aMWF between the splenium and genu of the corpus callosum.

We then performed subject‐level Spearman correlations between aMWF and dMRI metrics in WM, gCC, and sCC and calculated the mean across voxels from all participants (Figure 3; scatterplots depicting these correlations provided in Figure S4 and subject‐level correlations shown in Figure S5). All correlations were in the expected direction, with inverse correlations between aMWF and $\bar{D}$, *D*_⊥_, *K*_∥_, *D*_*a*_, and *D*_*e*,⊥_ and positive correlations with the remaining variables. We will examine these correlations based on the three primary hypotheses to facilitate interpretation of these results

Hypothesis 1 *Associations between aMWF and conventional DTI metrics in WM*.

Among traditional DTI metrics, the three directionally dependent diffusivities showed significant correlations with aMWF in the WM ROI (Figure 3A; positive correlations with FA and D_∥_ and an inverse correlation with *D*_⊥_, indicating reduced diffusion perpendicular to the axon in voxels with higher aMWF); these findings are consistent with prior research examining associations between DTI and conventional MWF [30, 31]. The strongest correlation was with FA (*ρ* = 0.56). $\bar{D}$ (which is rotationally invariant) showed no significant correlation with aMWF in WM. Together, these findings support Hypothesis 1 by demonstrating expected correlations between directionally dependent diffusion metrics and aMWF, indicating that aMWF is a suitable proxy for myelin content.

Hypothesis 2 *Associations between aMWF and kurtosis and biophysical metrics*.

Figure 3B displays associations between DKI, FBI, and FBWM metrics and aMWF within the WM ROI. All metrics had significant correlations in the expected direction with the exception of *K*_∥_, which was not significant. *f* showed the strongest association with aMWF in WM (*ρ* = 0.68), indicating that voxels with denser axonal packing had higher myelin content. Among DKI variables, $\bar{K}$ (*ρ* = 0.63) and *K*_⊥_ (*ρ* = 0.62) demonstrated the strongest associations with aMWF, indicating that non‐Gaussian diffusion closely corresponded with myelin content. The correlations for $\bar{K}$, *K*_⊥_, and *f* were substantially higher than the correlation with FA. Thus, these findings support Hypothesis 2 by demonstrating that advanced dMRI metrics accounting for limitations of conventional DTI display a stronger sensitivity to myelin content in WM.

Hypothesis 3 *Associations between aMWF and dMRI metrics in predefined WM ROIs*.

Figure 3C displays associations between all 16 dMRI metrics and aMWF for the sCC and gCC. Across almost all dMRI metrics, the sCC had stronger correlations with aMWF compared to gCC. For the sCC, the mean magnitude of correlation was|*ρ*| = 0.68, with 10 of the 16 correlations having|*ρ*| > 0.75. Very strong correlations (*ρ*s > 0.8) were present with FA (*ρ* = 0.88), *f* (*ρ* = 0.82), FAA (*ρ* = 0.82), MAA (*ρ* = 0.82), and *K*_⊥_ (*ρ* = 0.80). In comparison, the mean magnitude of correlation for the gCC was lower at|*ρ*| = 0.56, and only one correlation was higher than|*ρ*| > 0.75 (FA; *ρ* = 0.79). These results indicate that the associations between dMRI variables and myelin content are stronger in an earlier‐myelinating region with denser axonal packing and reduced fiber dispersion (i.e., the sCC) compared to a later myelinating region (gCC), consistent with the expected findings for Hypothesis 3.

An additional notable finding is that significant inverse correlations emerged between *K*_∥_ and aMWF within sCC (*ρ* = −0.68) and gCC (*ρ* = −0.47), whereas no significant correlation was present across the WM ROI. Thus, this directionally dependent metric may only reflect myelin content in circumscribed regions with denser axonal packing and myelination.

## 4 Discussion

This study examined associations between dMRI metrics and myelin content (using ViSTa aMWF as a surrogate marker) in healthy adults to determine myelin's influence on kurtosis and WM biophysical modeling parameters. Results replicated previous MWI studies [30, 31] in demonstrating that traditional DTI variables including *D*_∥_, *D*_⊥_, and FA corresponded with aMWF across WM regions. However, measures of kurtosis ($\bar{K}$ and *K*_⊥_) and the intra‐axonal compartment (*f*) demonstrated stronger associations with aMWF across WM than the conventional DTI metrics, indicating high sensitivity to myelin content regardless of region. Extra‐axonal metrics including *D*_*e*,∥_ and FAE also correlated with aMWF at a level comparable to conventional FA. Correlations were consistently stronger in magnitude within the sCC compared to the gCC and WM across all dMRI metric families.

These results demonstrate several important findings regarding myelin content and diffusion metrics associated with kurtosis and compartment‐specific biophysical modeling. Overall, aMWF corresponds with greater axonal packing (as reflected in *f*), coherence (FAA, MAA, and FAE), non‐Gaussian diffusion ($\bar{K}$), increased transverse heterogeneity (*K*_⊥_), and reduced transverse mobility in the extra‐axonal space (as reflected in *D*_e,⊥_). At the level of WM, the superiority of kurtosis (particularly $\bar{K}$ and *K*_⊥_) and *f* in tracking with aMWF compared to DTI metrics likely reflects two factors: (i) kurtosis metrics' sensitivity to microstructural complexity not captured by Gaussian models, and (ii) the compartmental specificity of FBI and FBWM, which arises from leveraging high and low *b*‐value dMRI data.

The spatial pattern showing the strongest associations in the sCC reflects how it is an early‐myelinating region with thicker axons, higher myelin content, reduced fiber‐orientation dispersion (i.e., greater alignment), and overall greater microstructural integrity compared to the gCC and other WM regions [10, 11, 12, 48]. Thus, the interpretation of dMRI metrics is associated with the regional degree of myelin content.

Notably, the discrepancy in correlation magnitude between the callosal and WM ROIs was not equivalent across variables for kurtosis and FBWM metrics. This was perhaps most dramatically demonstrated with K_∥_, which had substantial inverse correlations with aMWF in the sCC and gCC but no significant correlation across WM regions. This finding indicates that *K*_∥_ may only be sensitive to myelin content in WM regions with dense axon packing and low dispersion, and that higher orientation dispersion may disrupt the association with this metric. Likewise, the correlations between aMWF and both FAA and MAA were more than twice as high for the sCC compared to the WM ROI, indicating that the association between intra‐axonal anisotropy and myelin content also appears to be stronger in regions with lower fiber‐orientation dispersion and greater alignment.

Conversely, the differences in correlation between WM and the callosal ROIs were more modest for other kurtosis metrics ($\bar{K}$,*K*_⊥_, and KFA), f, and the extra‐axonal variables of *D*_*e*,∥_ and FAE. This finding indicates that these metrics may be less sensitive to regional characteristics of WM tracts such as fiber orientation and axon packing density and demonstrate more consistent associations with myelin content across regions. These findings are particularly notable for f, which represents the relative intra‐axonal water content. This metric displayed the strongest correlation with aMWF across WM (*ρ* = 0.68) and is the best proxy for overall myelin content among dMRI variables.

Methodologically, our results support utilizing multimodal protocols that combine ViSTa‐MWI with advanced dMRI metrics including DKI and FBWM to enhance the biological specificity of diffusion‐derived tissue properties. Practically, metrics including $\bar{K}$, *K*_⊥_, and *f* emerge as strongly myelin‐influenced candidates that are more sensitive than conventional DTI metrics (e.g., *D*_∥_, *D*_⊥_, and FA) in terms of sensitivity to myelin content across WM. Alternatively, *K*_∥_, FAA, and MAA demonstrate greater sensitivity to regional myelin content and may be valuable for observing differences between WM tracts. These characteristics may help to inform additional investigations into development, aging, and disorders impacting WM. They may also serve as quantitative benchmarks for longitudinal tracking or assessment of treatment response. However, this is contingent on their validation in a larger cohort to assess test–retest reliability and harmonization across scanners and sites.

Strengths of this study include the methodological rigor, including the use of advanced dMRI metrics and image processing tools. Methodologically, we mitigated CSF partial‐volume and misregistration by applying atlas‐based WM masking, diffusion‐space registration, and voxel filtering. The primary analysis of WM was also within deep white matter tracts that are reproducible across participants and cohorts. Although the JHU atlas does not include coverage for all cortical WM regions, it includes major tracts with a range of myelination to provide a preliminary analysis of associations between dMRI metrics and myelin content. We selected ROIs (sCC and gCC) in regions with well‐established differences in axon caliber and myelination for additional analysis, providing a strong foundation for evaluating how diffusion metrics scale with myelin content in regions with varying microstructural properties. The consistency between subjects and across metric families—kurtosis, intra‐axonal, and extra‐axonal—and across regions strengthens both internal validity and biological plausibility.

Limitations include how this analysis is an initial, small‐sample (*N* = 4), cross‐sectional study of healthy, mature WM; thus, our results should be interpreted only as within‐individual patterns. Second, aMWF is an indirect myelin marker whose absolute scaling (approximately 1/3 that of conventional MWF) depends on the details of the ViSTa sequence parameters [25]. We therefore emphasize associations rather than absolute quantification of myelin content. This smaller dynamic range may have contributed to the limited differences in aMWF observed across ROIs. Third, the use of DTI and DKI allows for generality of diffusion inference, regardless of tissue type. However, being general dMRI methods, DTI and DKI are not specific to mechanistic or biological processes. This is precisely the reason we have included more advanced high *b*‐value‐dependent biophysical modeling with FBI and FBWM to help isolate the myelin specificity of DTI and DKI metrics widely applied across a variety of clinically important pathologies. Fourth, FBI assumes properties for the dMRI signal and axon makeup, along with negligible exchange [13, 14, 15] and probably reflects only myelinated axons. Fifth, FBWM additionally assumes Gaussian extra‐axonal diffusion, but we have previously validated FBWM using triple diffusion encoding MRI (TDE), which does not specify any form for the extra‐axonal diffusion [49]. Sixth, we did not acquire repeated measurements at *b* = 6000 s/mm^2^ or noise‐only scans; therefore, absolute SNR could not be rigorously quantified in the coil‐combined magnitude images. The reported high‐*b* signal‐quality estimate was included to document that the dMRI data contained sufficient signal required for FBWM and was not used in statistical inference. Seventh, spatial autocorrelation may inflate voxel‐wise correlations by shared anatomy (e.g., both aMWF and anisotropy peak in tightly packed bundles). Eighth, we only chose two well‐defined ROIs from the corpus callosum with known cytoarchitectural and myelination differences for better clarity. We acknowledge that broader subject‐specific WM masks could have increased the dynamic range of aMWF values. However, broader masks may also introduce additional sources of variance, including partial‐volume effects, superficial WM registration error, crossing‐fiber architecture, and tract‐specific biological heterogeneity. Future work should extend these analyses to additional WM regions, including superficial regions, and evaluate whether associations vary by age or pathology. Finally, although we applied atlas‐based WM masking and diffusion‐based thresholds, residual CSF partial‐volume effects and registration imprecision at 2–3‐mm isotropic resolutions remain a distinct possibility. Although these constraints limit the scope of the analysis, they do not negate the key findings.

## 5 Conclusion

Our findings demonstrate that ViSTa‐MWI derived aMWF correlates strongly with DTI, DKI, and FBWM diffusion measures in healthy younger adults. Select DKI and FBWM metrics (e.g., $\bar{K}$, *K*_⊥_, and *f)* were more sensitive than standard DTI metrics to myelin content across WM, and other metrics (e.g., *K*_∥_, FAA, and MAA) showed strong regional differences regarding their associations with myelin content. Associations were consistently stronger in the sCC compared to the gCC, indicating that metrics better track with myelin content in regions with dense fiber packing, thicker myelin, and low fiber dispersion. We observed that higher myelin content co‐occurs with greater intra‐axonal water fraction and anisotropy, more hindered radial diffusion, and increased transverse microstructural complexity. These associations highlight the added specificity of DKI and FBWM over conventional DTI in capturing myelin‐related microstructural phenotyping. These advanced metrics might offer a starting point for future studies examining myelination and WM microstructure in other settings, such as across development, in healthy aging, and in clinical populations. By integrating ViSTa‐MWI with FBWM and DKI, researchers can more precisely track WM integrity across development, aging, and disease.

## Author Contributions

**Hunter G. Moss:** conceptualization, methodology, image processing, formal analysis, visualization, writing – original and final draft, review and editing. **Michael A. Sugarman:** visualization, writing – final draft, review and editing. **Jongho Lee:** conceptualization, methodology, formal analysis, writing – review and editing. **Andrew A. Chen:** formal analysis, statistical rigor, writing – review and editing. **Jens H. Jensen:** funding acquisition, resources, conceptualization, methodology, formal analysis, supervision, writing – review and editing. **Andreana Benitez:** funding acquisition, resources, conceptualization, methodology, formal analysis, supervision, writing – review and editing.

## Funding

This work is supported by the National Institutes of Health grant (R01AG054159).

## Conflicts of Interest

The authors declare no conflicts of interest.

## Supporting information


**Table S1:** Number of voxels for each subject in each ROI.
**Table S2:** Approximate SNR *b* = 6000 s/mm^2^ for each region of interest.
**Table S3:** The apparent myelin water fraction (%) across individuals and regions of interest.
**Table S4:** Subject‐level and summary statistics for dMRI metrics.
**Figure S1:** Axial image slices of the apparent myelin water fraction (aMWF), the complete set of dMRI tensor‐derived rotational invariants for diffusivity and kurtosis are presented, along with their respective anisotropies. The invariants for diffusivity are the mean, axial, and radial diffusivities ($\bar{D}$, $D_{\|}$, and $D_{⊥}$, respectively) and fractional anisotropy (FA). The analog invariants for kurtosis are the mean, axial, and radial kurtosis ($\bar{K}$, $K_{\|}$, and $K_{⊥}$, respectively) and kurtosis fractional anisotropy (KFA). All quantities are unitless except the diffusivities with units of μm^2^/ms.
**Figure S2:** The apparent myelin water fraction (aMWF) contrasted with biophysical modeling rotational invariants for the intra‐ and extra‐axonal spaces calculated using fiber ball imaging (FBI) and its association with the white matter model, FBWM. All quantities are dimensionless except for the diffusivities, which are measured in units of μm^2^/ms.
**Figure S3:** Violin plots illustrating all 16 dMRI metrics for individual subjects within each region of interest. The “white matter” ROI includes the entire JHU white matter atlas. The white dot indicates the median values, whereas the black bar within the distribution depicts the interquartile range. Figures on the right display box plots at the group level.
**Figure S4:** Scatterplots depicting the voxel‐wise correlations of each dMRI‐derived parameter analyzed with aMWF (from **Figure 3**). Each subject is represented with a different color. The Spearman's correlation represents the total correlation by pooling voxels across the four participants for each ROI.
**Figure S5:** Spearman correlation coefficients (*ρ*) for the apparent myelin water fraction (aMWF) with dMRI metrics for each subject. Correlations were calculated for each subject within the JHU WM atlas ROI as well as the genu and splenium of the corpus callosum. Positive and inverse correlations are marked by red and blue squares, respectively, with shading indicating the strength of the correlation. Most correlations remained statistically significant after 5000 block‐wise permutations to control for FWER (*p < 0.05; **p < 10^−3^), with those that did not survive indicated by more transparent squares.

## Data Availability

The research data are not shared.
