# Supplementary material for: The Association of Apparent Myelin Water Fraction With Diffusional Kurtosis and Biophysical Modeling Parameters
Source: NMR Biomed. 2026 Aug 27;39(10):e70377. doi: 10.1002/nbm.70377 (PMC13520807; doi:10.1002/nbm.70377)
Supplement: Supplementary file 1 [file NBM-39-e70377-s001.docx]

**Supplementary Material**

**Supplementary Table 1.** Number of voxels for each subject in each ROI.

| *Number of Voxels* | *White Matter* | *Genu* | *Splenium* |
| --- | --- | --- | --- |
| Subject 1 | 4479 | 277 | 386 |
| Subject 2 | 3197 | 175 | 207 |
| Subject 3 | 3961 | 237 | 325 |
| Subject 4 | 4196 | 245 | 373 |
| Average | 3958 ± 550 | 234 ± 43 | 323 ± 82 |

| The group average is ± Standard Error of the Mean  **Supplementary Table 2.** Approximate SNR *b* = 6000 s/mm^2^ for each region-of-interest. | | | |
| --- | --- | --- | --- |
| *ROI* | *White Matter* | *Genu* | *Splenium* |
| Subject 1 | 8 ± 2 [2, 13] | 6 ± 1 [3, 9] | 8 ± 1 [5, 12] |
| Subject 2 | 9 ± 3 [2, 17] | 6 ± 1 [2, 9] | 10 ± 2 [5, 16] |
| Subject 3 | 8 ± 2 [2, 14] | 6 ± 1 [2, 8] | 8 ± 1 [2, 12] |
| Subject 4 | 8 ± 2 [2, 14] | 6 ± 1 [3, 8] | 7 ± 1 [2, 12] |
| Average | 8 ± 1 | 6 ± 1 | 8 ± 1 |
| Values are Mean ± Standard Deviation [Min, Max] while the group average is ± Standard Error of the Mean. | | | |

| **Supplementary Table 3.** The apparent myelin water fraction (%) across individuals and regions-of-interest. | | | |
| --- | --- | --- | --- |
| *ROI* | *White Matter* | *Genu* | *Splenium* |
| Subject 1 | 5.12 ± 1.15 [1.67, 8.75] | 5.05 ± 1.12 [2.37, 7.53] | 6.10 ± 1.21 [3.09, 8.75] |
| Subject 2 | 4.52 ± 1.09 [1.54, 7.67] | 4.28 ± 0.84 [2.19, 5.97] | 5.37 ± 1.08 [3.04, 7.59] |
| Subject 3 | 4.41 ± 1.06 [1.71, 7.53] | 4.61 ± 0.99 [2.41, 6.95] | 4.86 ± 1.28 [2.34, 7.53] |
| Subject 4 | 5.31 ± 1.20 [1.82, 8.13] | 5.92 ± 0.97 [2.61, 7.64] | 5.49 ± 1.32 [2.56, 8.13] |
| Average | 4.84 ± 0.44 | 4.97 ± 0.71 | 5.45 ± 0.51 |
| Values are Mean ± Standard Deviation [Min, Max] while the group average is ± Standard Error of the Mean. | | | |

**Supplementary Table 4.** Subject-level and summary statistics for dMRI metrics.

| **Diffusivity** | | | | |
| --- | --- | --- | --- | --- |
| ***Metric*** | ***Individual*** | ***White Matter*** | ***Genu*** | ***Splenium*** |
| $\bar{D}$ | Subject 1 | 0.89 ± 0.09 [0.70, 1.49] | 0.92 ± 0.09 [0.80, 1.41] | 0.94 ± 0.14 [0.75, 1.49] |
|  | Subject 2 | 0.85 ± 0.12 [0.63, 1.50] | 0.95 ± 0.14 [0.79, 1.48] | 0.91 ± 0.15 [0.70, 1.50] |
|  | Subject 3 | 0.85 ± 0.09 [0.66, 1.50] | 0.94 ± 0.12 [0.79, 1.45] | 0.92 ± 0.13 [0.72, 1.50] |
|  | Subject 4 | 0.87 ± 0.09 [0.68, 1.48] | 0.95 ± 0.13 [0.76, 1.46] | 0.93 ± 0.12 [0.76, 1.48] |
|  | **Average** | **0.86 ± 0.01** | **0.94 ± 0.01** | **0.93 ± 0.01** |
| $D_{\vert\vert}$ | Subject 1 | 1.37 ± 0.25 [0.88, 2.56] | 1.48 ± 0.30 [0.95, 2.20] | 1.70 ± 0.31 [1.03, 2.56] |
|  | Subject 2 | 1.25 ± 0.25 [0.78, 2.62] | 1.42 ± 0.35 [0.96, 2.36] | 1.62 ± 0.41 [0.86, 2.62] |
|  | Subject 3 | 1.26 ± 0.26 [0.79, 2.49] | 1.51 ± 0.33 [0.96, 2.45] | 1.55 ± 0.39 [0.90, 2.49] |
|  | Subject 4 | 1.30 ± 0.25 [0.83, 2.75] | 1.64 ± 0.25 [1.03, 2.38] | 1.57 ± 0.38 [0.89, 2.75] |
|  | **Average** | **1.30 ± 0.03** | **1.51 ± 0.05** | **1.61 ± 0.03** |
| $D_{\perp}$ | Subject 1 | 0.64 ± 0.13 [0.21, 1.36] | 0.64 ± 0.15 [0.29, 1.25] | 0.56 ± 0.18 [0.21, 1.15] |
|  | Subject 2 | 0.64 ± 0.16 [0.21, 1.44] | 0.71 ± 0.18 [0.26, 1.34] | 0.55 ± 0.20 [0.21, 1.20] |
|  | Subject 3 | 0.64 ± 0.13 [0.17, 1.40] | 0.66 ± 0.17 [0.26, 1.23] | 0.61 ± 0.20 [0.17, 1.31] |
|  | Subject 4 | 0.65 ± 0.14 [0.20, 1.32] | 0.60 ± 0.18 [0.28, 1.20] | 0.62 ± 0.21 [0.20, 1.31] |
|  | **Average** | **0.64 ± 0.00** | **0.65 ± 0.02** | **0.58 ± 0.02** |
| FA | Subject 1 | 0.45 ± 0.15 [0.06, 0.88] | 0.47 ± 0.18 [0.10, 0.84] | 0.62 ± 0.15 [0.23, 0.88] |
|  | Subject 2 | 0.41 ± 0.17 [0.02, 0.88] | 0.40 ± 0.18 [0.05, 0.86] | 0.58 ± 0.21 [0.04, 0.88] |
|  | Subject 3 | 0.41 ± 0.16 [0.02, 0.90] | 0.47 ± 0.19 [0.05, 0.85] | 0.52 ± 0.22 [0.03, 0.90] |
|  | Subject 4 | 0.43 ± 0.16 [0.02, 0.89] | 0.56 ± 0.15 [0.11, 0.83] | 0.52 ± 0.23 [0.02, 0.89] |
|  | **Average** | **0.42 ± 0.01** | **0.48 ± 0.03** | **0.56 ± 0.02** |
| **Kurtosis** | | | | |
| ***Metric*** | ***Individual*** | ***White Matter*** | ***Genu*** | ***Splenium*** |
| $\bar{K}$ | Subject 1 | 0.99 ± 0.14 [0.46, 1.48] | 0.95 ± 0.12 [0.60, 1.24] | 1.10 ± 0.13 [0.70, 1.44] |
|  | Subject 2 | 1.04 ± 0.18 [0.37, 1.57] | 0.95 ± 0.13 [0.56, 1.22] | 1.14 ± 0.17 [0.67, 1.57] |
|  | Subject 3 | 1.00 ± 0.16 [0.27, 1.43] | 0.96 ± 0.14 [0.50, 1.24] | 1.03 ± 0.15 [0.65, 1.42] |
|  | Subject 4 | 1.04 ± 0.17 [0.36, 1.69] | 0.98 ± 0.13 [0.54, 1.26] | 1.07 ± 0.19 [0.63, 1.69] |
|  | **Average** | **1.02 ± 0.01** | **0.96 ± 0.01** | **1.09 ± 0.02** |
| $K_{\vert\vert}$ | Subject 1 | 0.70 ± 0.16 [0.15, 1.46] | 0.64 ± 0.15 [0.24, 1.06] | 0.58 ± 0.17 [0.15, 1.19] |
|  | Subject 2 | 0.77 ± 0.17 [0.18, 1.51] | 0.66 ± 0.17 [0.18, 1.23] | 0.62 ± 0.19 [0.35, 1.28] |
|  | Subject 3 | 0.74 ± 0.18 [0.20, 1.57] | 0.61 ± 0.15 [0.25, 1.05] | 0.64 ± 0.18 [0.20, 1.19] |
|  | Subject 4 | 0.75 ± 0.18 [0.22, 1.50] | 0.57 ± 0.10 [0.32, 1.03] | 0.61 ± 0.17 [0.22, 1.00] |
|  | **Average** | **0.74 ± 0.01** | **0.62 ± 0.02** | **0.61 ± 0.01** |
| $K_{\perp}$ | Subject 1 | 1.44 ± 0.39 [0.39, 3.33] | 1.45 ± 0.42 [0.66, 3.12] | 1.84 ± 0.48 [0.39, 3.19] |
|  | Subject 2 | 1.41 ± 0.41 [0.38, 3.73] | 1.41 ± 0.43 [0.65, 2.71] | 1.87 ± 0.63 [0.64, 3.73] |
|  | Subject 3 | 1.41 ± 0.40 [0.32, 3.49] | 1.52 ± 0.45 [0.61, 2.82] | 1.64 ± 0.61 [0.65, 3.49] |
|  | Subject 4 | 1.45 ± 0.42 [0.30, 3.96] | 1.56 ± 0.39 [0.55, 2.79] | 1.72 ± 0.73 [0.62, 3.96] |
|  | **Average** | **1.43 ± 0.01** | **1.49 ± 0.03** | **1.77 ± 0.05** |
| KFA | Subject 1 | 0.49 ± 0.11 [0.10, 0.81] | 0.51 ± 0.13 [0.21, 0.78] | 0.53 ± 0.12 [0.21, 0.79] |
|  | Subject 2 | 0.47 ± 0.14 [0.05, 0.87] | 0.44 ± 0.15 [0.12, 0.79] | 0.52 ± 0.14 [0.16, 0.79] |
|  | Subject 3 | 0.48 ± 0.13 [0.08, 0.87] | 0.47 ± 0.14 [0.12, 0.78] | 0.51 ± 0.15 [0.12, 0.80] |
|  | Subject 4 | 0.48 ± 0.13 [0.07, 0.87] | 0.51 ± 0.13 [0.17, 0.79] | 0.48 ± 0.15 [0.09, 0.78] |
|  | **Average** | **0.48 ± 0.01** | **0.48 ± 0.02** | **0.51 ± 0.01** |
| **Intra-axonal** | | | | |
| ***Metric*** | ***Individual*** | ***White Matter*** | ***Genu*** | ***Splenium*** |
| *f* | Subject 1 | 0.54 ± 0.09 [0.21, 0.79] | 0.50 ± 0.09 [0.21, 0.69] | 0.62 ± 0.07 [0.38, 0.79] |
|  | Subject 2 | 0.55 ± 0.13 [0.15, 0.90] | 0.49 ± 0.11 [0.17, 0.66] | 0.63 ± 0.11 [0.27, 0.90] |
|  | Subject 3 | 0.54 ± 0.11 [0.14, 0.79] | 0.52 ± 0.11 [0.18, 0.72] | 0.57 ± 0.10 [0.22, 0.79] |
|  | Subject 4 | 0.56 ± 0.12 [0.14, 0.84] | 0.54 ± 0.09 [0.24, 0.69] | 0.57 ± 0.12 [0.21, 0.84] |
|  | **Average** | **0.55 ± 0.01** | **0.51 ± 0.01** | **0.60 ± 0.02** |
| *D_a_* | Subject 1 | 2.58 ± 0.40 [1.06, 6.29] | 2.77 ± 0.53 [1.71, 6.29] | 2.63 ± 0.58 [1.47, 4.41] |
|  | Subject 2 | 2.51 ± 0.76 [0.73, 9.57] | 2.90 ± 0.75 [1.67, 6.83] | 2.52 ± 0.54 [1.51, 4.84] |
|  | Subject 3 | 2.48 ± 0.50 [0.60, 6.91] | 2.81 ± 0.61 [1.74, 6.79] | 2.61 ± 0.56 [1.40, 5.02] |
|  | Subject 4 | 2.52 ± 0.48 [0.78, 7.25] | 2.61 ± 0.61 [1.41, 5.13] | 2.72 ± 0.59 [1.53, 6.57] |
|  | **Average** | **2.52 ± 0.02** | **2.77 ± 0.06** | **2.62 ± 0.04** |
| FAA | Subject 1 | 0.60 ± 0.14 [0.10, 0.91] | 0.63 ± 0.14 [0.15, 0.88] | 0.74 ± 0.11 [0.33, 0.89] |
|  | Subject 2 | 0.53 ± 0.15 [0.09, 0.88] | 0.60 ± 0.17 [0.14, 0.88] | 0.68 ± 0.16 [0.16, 0.88] |
|  | Subject 3 | 0.54 ± 0.15 [0.10, 0.91] | 0.66 ± 0.16 [0.16, 0.89] | 0.64 ± 0.18 [0.11, 0.89] |
|  | Subject 4 | 0.55 ± 0.14 [0.10, 0.90] | 0.71 ± 0.10 [0.21, 0.86] | 0.64 ± 0.18 [0.18, 0.90] |
|  | **Average** | **0.55 ± 0.01** | **0.65 ± 0.02** | **0.67 ± 0.02** |
| MAA | Subject 1 | 0.25 ± 0.06 [0.09, 0.44] | 0.28 ± 0.08 [0.10, 0.44] | 0.32 ± 0.06 [0.15, 0.43] |
|  | Subject 2 | 0.21 ± 0.06 [0.06, 0.41] | 0.25 ± 0.08 [0.09, 0.41] | 0.28 ± 0.07 [0.09, 0.40] |
|  | Subject 3 | 0.22 ± 0.06 [0.08, 0.44] | 0.27 ± 0.08 [0.10, 0.42] | 0.27 ± 0.08 [0.11, 0.42] |
|  | Subject 4 | 0.23 ± 0.06 [0.08, 0.42] | 0.30 ± 0.05 [0.12, 0.39] | 0.27 ± 0.08 [0.08, 0.42] |
|  | **Average** | **0.23 ± 0.01** | **0.27 ± 0.01** | **0.28 ± 0.01** |
| **Extra-axonal** | | | | |
| ***Metric*** | ***Individual*** | ***White Matter*** | ***Genu*** | ***Splenium*** |
| $\bar{D}_{e}$ | Subject 1 | 0.93 ± 0.10 [0.69, 1.64] | 0.94 ± 0.10 [0.78, 1.30] | 1.04 ± 0.16 [0.76, 1.64] |
|  | Subject 2 | 0.90 ± 0.12 [0.67, 2.10] | 0.97 ± 0.14 [0.76, 1.44] | 1.07 ± 0.23 [0.74, 2.10] |
|  | Subject 3 | 0.90 ± 0.10 [0.64, 1.58] | 0.97 ± 0.12 [0.78, 1.42] | 1.03 ± 0.17 [0.72, 1.58] |
|  | Subject 4 | 0.93 ± 0.11 [0.71, 1.77] | 1.05 ± 0.14 [0.80, 1.50] | 1.02 ± 0.17 [0.75, 1.77] |
|  | **Average** | **0.91 ± 0.01** | **0.98 ± 0.02** | **1.04 ± 0.01** |
| $D_{\vert\vert,e}$ | Subject 1 | 1.25 ± 0.29 [0.79, 3.21] | 1.32 ± 0.38 [0.88, 2.67] | 1.60 ± 0.53 [0.83, 3.21] |
|  | Subject 2 | 1.22 ± 0.34 [0.75, 3.55] | 1.23 ± 0.32 [0.83, 2.87] | 1.71 ± 0.72 [0.81, 3.55] |
|  | Subject 3 | 1.20 ± 0.31 [0.76, 3.52] | 1.30 ± 0.32 [0.85, 2.68] | 1.55 ± 0.62 [0.80, 3.52] |
|  | Subject 4 | 1.26 ± 0.35 [0.80, 3.49] | 1.57 ± 0.48 [0.85, 3.00] | 1.52 ± 0.64 [0.80, 3.49] |
|  | **Average** | **1.23 ± 0.01** | **1.36 ± 0.08** | **1.60 ± 0.04** |
| $D_{\perp,e}$ | Subject 1 | 0.77 ± 0.13 [0.25, 1.54] | 0.75 ± 0.13 [0.36, 1.25] | 0.76 ± 0.21 [0.25, 1.54] |
|  | Subject 2 | 0.74 ± 0.13 [0.29, 1.68] | 0.84 ± 0.15 [0.32, 1.35] | 0.75 ± 0.26 [0.29, 1.68] |
|  | Subject 3 | 0.76 ± 0.11 [0.19, 1.49] | 0.81 ± 0.15 [0.37, 1.40] | 0.76 ± 0.22 [0.19, 1.49] |
|  | Subject 4 | 0.76 ± 0.13 [0.25, 1.64] | 0.79 ± 0.19 [0.35, 1.40] | 0.77 ± 0.22 [0.25, 1.64] |
|  | **Average** | **0.75 ± 0.01** | **0.80 ± 0.02** | **0.76 ± 0.00** |
| FAE | Subject 1 | 0.30 ± 0.15 [0.02, 0.91] | 0.33 ± 0.20 [0.05, 0.83] | 0.42 ± 0.23 [0.05, 0.91] |
|  | Subject 2 | 0.31 ± 0.17 [0.02, 0.91] | 0.23 ± 0.15 [0.03, 0.88] | 0.43 ± 0.27 [0.06, 0.91] |
|  | Subject 3 | 0.28 ± 0.16 [0.02, 0.94] | 0.28 ± 0.17 [0.04, 0.84] | 0.38 ± 0.25 [0.04, 0.94] |
|  | Subject 4 | 0.30 ± 0.17 [0.02, 0.92] | 0.39 ± 0.21 [0.03, 0.86] | 0.36 ± 0.26 [0.05, 0.92] |
|  | **Average** | **0.30 ± 0.01** | **0.31 ± 0.03** | **0.40 ± 0.02** |
| Values are Mean ± Standard Deviation [Min, Max] while the group average is ± Standard Error of the Mean. Diffusivities are in units of [μm^2^/ms]. | | | | |

**Supplementary Figure 1.** Axial image slices of the apparent myelin water fraction (aMWF), the complete set of dMRI tensor-derived rotational invariants for diffusivity and kurtosis are presented, along with their respective anisotropies. The invariants for diffusivity are the mean, axial, and radial diffusivities ($\bar{D}$, $D_{||}$, and $D_{\perp}$, respectively) and fractional anisotropy (FA). The analog invariants for kurtosis are the mean, axial, and radial kurtosis ($\bar{K}$, $K_{||}$, and $K_{\perp}$, respectively) and kurtosis fractional anisotropy (KFA). All quantities are unitless except the diffusivities with units of μm^2^/ms.

**Supplementary Figure 2.** The apparent myelin water fraction (aMWF) contrasted with biophysical modeling rotational invariants for the intra- and extra-axonal spaces calculated using fiber ball imaging (FBI) and its association with the white matter model, FBWM. All quantities are dimensionless except for the diffusivities, which are measured in units of μm^2^/ms.

**Supplementary Figure 3.** Violin plots illustrating all 16 dMRI metrics for individual subjects within each region of interest. The “white matter” ROI includes the entire JHU white matter atlas. The white dot indicates the median values, while the black bar within the distribution depicts the interquartile range. Figures on the right display box plots at the group level.

**Supplementary Figure 4.** Scatterplots depicting the voxel-wise correlations of each dMRI-derived parameter analyzed with aMWF (from **Figure 3**). Each subject is represented with a different color. The Spearman’s correlation represents the total correlation by pooling voxels across the four participants for each ROI.

**Supplementary Figure 5.** Spearman correlation coefficients (*ρ*) for the apparent myelin water fraction (aMWF) with dMRI metrics for each subject. Correlations were calculated for each subject within the JHU WM atlas ROI as well as the genu and splenium of the corpus callosum. Positive and inverse correlations are marked by red and blue squares, respectively, with shading indicating the strength of the correlation. Most correlations remained statistically significant after 5000 block-wise permutations to control for FWER (* *p* < 0.05; ** *p* < 10^-3^), with those that did not survive indicated by more transparent squares.
